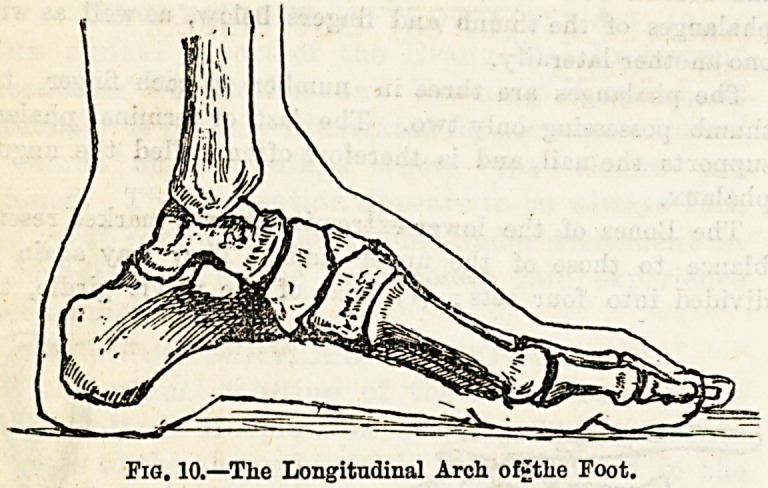# The Hospital Nursing Supplement

**Published:** 1895-02-02

**Authors:** 


### "The Hospital" Nursing Mirror.

Being the Extra Nursing Supplement of "The Hospital" Newspaper.

[Contributions for this Supplement should be addressed to the Editor, The Hospital, 428, Strand, London, W.C., and should have the word "Nursing" plainly written in left-hand top corner of the envelope.]

### News from the Nursing World.

### PRINCESS BEATRICE AT RYDE.

H.R.H. Princess Beatrice attended the annual meeting of the Ryde District Nursing Association on January 23rd. Her Royal Highness has always taken great interest in this useful society, of which she is president. The meeting was held in Ryde Town Hall, and was well attended.

### ST. THOMAS'S HOSPITAL.

An excellent programme was arranged for the nurses' annual concert on January 24th by the students of St. Thomas's Hospital, who are to be congratulated on the success of a really brilliant entertainment. Many old workers, members of the visiting staff, and guests from other hospitals, were noticeable amongst the large and appreciative audience which filled the hall. Dr. Toller's exquisite violin solos were enthusiastically encored, and Madame Gomez, Miss Bristowe, Miss Elsie Mackenzie, and Mr. Devonshire were amongst the singers who kindly contributed to the success of this pleasant evening. The whistling solos of Mr. Mackay were very clever, and the musical sketches of Mr. Dudley Causton were as amusing as they were varied and original. Before the singing of "God Save the Queen" concluded the concert the treasurer made a short speech, in which he pleaded most eloquently for financial support for the hospital, to enable wards now empty to be peopled by patients, and also for funds to remove the many anxieties which at present made his official position one of grave responsibility.

### AT THE NEW HOSPITAL FOR WOMEN.

Many qualified medical women and other guests took tea at the new Hospital for Women, in Euston Road, on the 24th ult. The pretty and well-ordered institution was inspected by a number of visitors, who did not fail to note many admirable features, and foremost of all the comfortable and contented appearance of the patients. "And what about your convalescent home?" was asked by a stranger; "Is it a success?" The reply came from the occupant of one of the beds —"I should not be alive now but for 'The Cottage,'" interposed the little woman. "It's just fine to be there; you're considered in everything, and taken such care of. Nothing's made a trouble of there; think of that, ma'am, when you're weak and ill, and afraid of being a burden. Then the place is really countrified —thatched cottages, and green fields and hills. I'm going there again soon to get up my strength before returning home; but I'm wonderful comfortable here, too, that I am!"

### FAVOURED FEVER NURSES.

"She might do for a fever nurse," used frequently to be said of a woman who had no qualifications, yet every necessity, for earning her own living. But the day is almost past when "anyone" was made welcome in an isolation hospital. Not only is good training assured to intelligent, educated women, but the accommodation as well as the pay under many of the sanitary authorities compare most favourably with that of some general hospitals. At the Western Hospital, at Fulham (Metropolitan Asylums Board), pleasant sitting and mess rooms are provided for the nursing staff, and separate sleeping rooms for each. A good piano shows that recreation is duly considered, and regular courses of lectures are given by the Medical Superintendent. Besides receiving good salaries, nurses in fever hospitals are exempt from laundry expenses, all washing being done in the separate "staff" department. These privileges make an infectious hospital very far from the "dreadful place" to work in which it is even now considered by those who have never been inside the walls. Nurses realising the valuable experience to be gained in fever wards, and entering them at a suitable age, neither require nor desire the commiseration the public bestowsupon them.

### ANOTHER NURSES' HOME.

It is nearly a quarter of a century since the three years' course of training found favour in St. Pancras Infirmary, Highgate Hill, and recently a fine nurses' home has been added to the building, which adjoins Waterlow Park. By this means accommodation has been secured for an increased number of probationers, as well as for a second night superintendent and for sundry offices. The single bed-rooms are plainly but suitably furnished, and the bath-rooms and box-rooms seem convenient, although the provision of cupboards and presses appears somewhat unduly restricted. For the seven charge nurses a cosy littte sitting-room is set apart, whilst the recreation-room, common to the whole staff, is a really delightful apartment. It has been furnished with common sense, as well as with taste, and by certain ingeniously contrived panels it can be divided into two rooms either for classes or the medical officer's courses of lectures. When some liberal-minded Guardians add the well-stocked bookshelves, without which no modern nurses' home is complete, the St. Pancras' staff will have little left to wish for. The kindly spirit and pleasant harmony which pervades this infirmary show how cordially Miss Moir's labours in the nursing department are aided and encouraged by the medical superintendent.

### HELPS OR HINDRANCES?

"They want a woman on the Board" is a natural remark when Guardians are heard to order a large supply of corsets "all the same size" for female inmates of various ages and shapes. Women Guardians, however, do not as yet seem quite to understand that however valuable they may prove in certain practical matters concerning the personal comfort of paupers, they, like every one else, need to exercise infinite discretion and tact whilst holding office. When we read of a lady maintaining that an untrained matron is competent to supervise trained nurses and to instruct regular probationers, and when another considers herself better fitted to select nurse candidates than a matron who has had many years experience, we realise that lady Guardians are not unmitigated blessings. They need educating for the posts they aspire to fill just as much as those workers they sometimes severely criticise, and until they add discretion to zeal they are likely to mar rather than to make needful reforms.

### DEACONESSES AT TOTTENHAM.

The annual report of the Evangelical Protestant Deaconesses' Institution and Training Hospital, at Tottenham, is prettily illustrated by views of some of the wards and buildings where the sisters are stationed. The institution appears to be always full, and a great variety of cases come under treatment in the hospital, where the deaconesses gain experience under trained nurses.

### FAITHFUL SERVICES ACKNOWLEDGED.

At the annual meeting of the subscribers to the Ealing Cottage Hospital on January 19th, reference was made to the changes which have occurred in the nursing staff during the past year. Miss Reid, who as matron served the hospital well from its opening, twenty-three years ago, having resigned, she was replaced by Miss Titherington, trained at the Middlesex Hospital. Appreciative mention was made of Miss Reid's services, and in addition to the sum of £150 which was collected and presented to her on her retirement, it was unanimously agreed that a pension of £25 a year should be drawn from the reserve fund of the hospital for her benefit.

### WANTED—A NIGHT NURSE.

The Coventry Guardians are still divided as to the necessity for a night nurse for their infirmary, some of the Guardians seeming content with a pauper wardsman in that capacity. An inquest or perhaps a special recommendation from the Local Government Board may possibly arouse to a more humane view of their responsibilities these guardians of the helpless poor. The Medical Inspector made an eloquent appeal at a recent meeting, asking the Guardians whether they would like to find themselves or their relations in incompetent hands in their last hours. He also remarked, in connection with pauper helpers, that first-rate or reliable people would not be likely to remain inmates of workhouses.

### DISTRICT NURSES AT PERTH.

There was a very large attendance at the Commissioners' Hall on the occasion of the annual meeting of the Perth Sick Poor Nursing Society, which continues to cover a large and increasing field of usefulness. The nursing of out-door patients of the infirmary is also undertaken by the district nurses by special arrangement with the directors of the institution. The Ladies' Needlework Guild has proved most helpful, and a daily supply of soup for special cases is another valuable branch of assistance rendered to the association. The Superintendent, Miss Beatrice Graeme and Nurse M'Queen are both Queen's Nurses, and appear to be much valued in the neighbourhood.

### INCOMPETENT CARETAKERS.

"Perhaps they had been partially neglected in the past," remarked a guardian at Liskeard the other day with reference to eight baby inmates whom the matron's numerous duties prevented her "looking after" personally. An incapable old woman of seventy has been in charge of these infants, assisted by an imbecile girl. The impossibility of her other duties permitting any workhouse matron to give due attention to so many children was apparently obvious to only a small minority of the Guardians, yet a proposal to employ a paid nurse for the unfortunate infants was negatived. The inspector's report shows that the children at present are improperly fed and unhealthy looking. The matron declined, as reported by the local press, to admit any responsibility for their feeding. She of course cannot ensure the daily allowance of milk being justly administered. Doubtless the matron herself has done her best for the babies, but the quality of the pauper help available is sufficient proof of the faultiness of the system which condemns to such environment sickly children whose mental as well as physical development calls for the constant supervision of intelligent, high-principled women.

### SICK BABIES AT FLORENCE.

A hospital for sick babies was opened in January at Florence. It is built in two pavilions, one of which consists of four wards for general diseases; and separated by two corridors stands the second block, containing six wards devoted to babies suffering from syphilis, thrush, or ophthalmia. So-called "incubating rooms" form a special feature of this new hospital, and in them feeble and prematurely born infants are kept in a temperature of 68 deg. to 86 deg. Fahr.

### TRAINING AT MONTREAL.

Four years ago a training school for nurses was organised in connection with the General Hospital at Montreal, and already a two years' course has been completed by seventy-two graduates. Three examinations take place during the training, and classes are held by the Lady Superintendent, lectures being given during the winter months by the visiting staff. Candidates have to serve a two months' probation before being accepted for training, and the school seems to be a promising one.

### SHORT ITEMS.

The annual meeting of the Ashburton District Nurses' Association took place last month. The Hon. Secretary expressed appreciation of the useful work done by Nurse Salter.—The Duchess of Abercorn presided last week at the annual meeting of the Londonderry Nursing Association, when several interesting speeches were made.—The suggestion that a trained nurse should be employed at Penzance, where the "pauper ward assistants are old and of a poor type," has not been followed by the Guardians, the majority of whom apparently fail to realise the inadequacy of their present provision for the care of the sick and infirm.—The annual meeting of the Tunstall Town Nursing and Samaritan Society was held recently, very satisfactory reports being given of both branches of the work.—Miss Pittendriegh, of Dundee, has been appointed district nurse to the Dysart Victoria Nursing Association; the work of Miss Rose (Queen's Nurse) appears to have been highly appreciated during the weeks in which she was temporarily in charge at Dysart.—The Mayor presided at the annual meeting of the Leeds District Nursing Association which is now affiliated with the Q.V.J.I.—Miss Tulloch, Superintendent of a private hospital in Howard Street, Belfast, tells us that she has now taken over the hospital on her own account, and patients can be attended by their own medical attendants.

### Elementary Anatomy and Surgery for Nurses.

By W. McAdam Eccles, M.B., B.S., F.R.C.S., Lecturer to Nurses, West London Hospital.

### IV.—THE OSSEOUS SYSTEM.—(*Continued*).

### The Bones of the Upper and Lower Extremities.

The Bones of the upper extremity fall conveniently into four groups: (1) Those of the shoulder girdle, the clavicle and the scapula; (2) that of the arm proper, the humerus; (3) those of the forearm, the radius and the ulna; and (4) those of the wrist and hand, the carpus, the metacarpus, and the phalanges.

The clavicle, or collar-bone (see Fig. 8) is supposed to resemble a key in shape. It presents two curves, and articulates internally with the upper piece of the sternum, and externally with the acromion process of the scapula. Its chief function is to keep the shoulders back, and to form an, attachment for the upper limb to the trunk.

The scapula, or shoulder-blade, is a broad, flat bone triangular in shape, with three well-marked processes—(1) the spine, ending in (2) the acromion process, and lastly (3) the coracoid process. The acromion process articulates with the outer end of the clavicle. At the upper and external angle of the scapula, overhung by the acromion and coracoid processes, is a shallow depression called the glenoid cavity, with which the head of the humerus articulates. The scapula is very freely movable, being attached to the trunk chiefly by muscles.

The humerus is a long bone, and consists of a shaft and two extremities, the upper being called the head. This is a hemispherical prominence which articulates with the glenoid cavity of the scapula. Below it are the two tuberosities, the external being the larger, and between the two is a groove for the long tendon of the biceps muscle. The humerus has two necks, one the anatomical—the groove between the head and the tuberosities—the other the constricted part below the tuberosities. The shaft has running posteriorly from within downwards and outwards a depression styled the musculo-spinal groove, in which lie an artery and nerve. The lower extremity is expanded and shows two fossæ or pits, one in front named the coronoid, the other, deeper, behind, the olecranon fossa. In addition there are two condyles, the internal being the larger, and behind it runs a nerve which when struck gives rise to great pain, and is often called the "funny bone;" and two articular surfaces, the outer for the radius, the inner for the ulna. Over the extremities of the humerus, which are separate from the shaft in infant life, can be seen numerous foramina or holes for the entrance of blood vessels into the substance of the bone; the shaft, moreover, presents a single large foramen for the nutrient artery of that part of the bone. This arrangement of bloodvessels holds good in most of the long bones.

The radius, the outer bone of the forearm, is also a long bone with a shaft and two extremities, the upper being styled the head, articulating both with the humerus and ulna. Below this is the neck, presenting on the inner side the prominence termed the tubercle. The lower extremity has externally a projection called the styloid process, posteriorly grooves for tendons, or "leaders," and beneath articular surfaces for two of the wrist bones. This part of the radius also again articulates with the lower end of the ulna.

The ulna, the inner bone of the forearm, has at its upper end two outstanding points, the coronoid process anteriorly, and the olecranon process posteriorly, the latter forming the prominence of the elbow. Between these two is a deep cavity which articulates with the humerus, and external to them a much shallower depression to receive the head of the radius. The shaft of the ulna presents a well-marked ridge posteriorly, which can be easily felt beneath the skin. The lower end is called the head, and to it is attached below the styloid process.

The carpus, or the bones of the wrist, consists of eight separate bones in two rows of four each, all articulating with neighbouring bones.

The metacarpus is made up of five bones forming the bony framework of the palm of the hand; they articulate with the second row of the carpus above, and with the first phalanges of the thumb and fingers below, as well as with one another laterally.

The phalanges are three in number to each finger, the thumb possessing only two. The last or terminal phalanx supports the nail, and is therefore often called the ungual phalanx.

The Bones of the lower extremity show a marked resemblance to those of the upper limb. They may again be divided into four sets: (1) That of the pelvic girdle, the

Fig. 8.—Bones of the Upper Extremity.

Fig. 9.—Bones of the Lower Extremity.

os innominatum, or hip-bone; (2) that of the thigh, the femur; (3) those of the leg, the tibia with the patella, and the fibula; (4) those of the ankle and foot, the tarsus, the metatarsus, and the phalanges.

The os innominatum or hip-bone (see Fig. 9), together with its fellow of the opposite side, and the sacrum, already spoken of, form the pelvis—a basin-like cavity at the lower part of the abdomen. The hip-bone is an irregularly shaped bone, consisting in early life of three bones which later become fused together. The three parts are respectively called the ilium, the ischium, and the os pubis. On the ischium is a strong, well marked prominence, termed the tuberosity, upon which we sit. Another well marked prominence situated in front on the ilium is called the anterior superior spine. The two pubic bones meet in front, forming the symphysis pubis, and the anterior part of the circle of the pelvis. Between the os pubis and ischium is a large aperture called the thyroid or obturator foramen. Externally there is a deep cavity, the margin of which is incomplete below. This is the acetabulum, and receives the head of the femur.

Fig. 10.—The Longitudinal Arch of the Foot.

The femur, or thigh-bone—the longest and strongest bone of the extremities—has a spherical head at the upper end on which is a depression for a ligament. The neck is just below the head, and there may be seen two marked projections, the trochanter major and minor. The shaft is prismatic in form, having a sharp ridge behind. The lower extremity is expanded, and presents two condyles which articulate with the upper end of the tibia, and with the patella The flat surface behind these is called the popliteal aspect of the femur.

The tibia is the shin-bone, and the larger and inner bone of the leg. The upper extremity is enlarged, forming two tuberosities with articular surfaces for the condyles of the femur, and a small facet on the outer side for the fibula. Below the head in front is the tubercle, and running down from this is a ridge called the shin, which is subcutaneous. At the lower end is a projection, the internal malleolus, or ankle.

The patella, or knee-pan, corresponds to the olecranous process of the ulna. It is a sesamoid bone, that is, a bone developed in the tendon of a muscle. The patella articulates with the condyles of the femur.

The fibula, the outer bone of the leg, is a slender long bone having a head at the upper end, and a pointed process below called the external malleolus. A rough triangular surface above this is subcutaneous. The fibula articulates above with the tibia, and below again with the tibia and also with the astragalus, an important bone of the tarsus.

The tarsus consists of seven bones in all. One, the astragalus, fits in between the two malleoli; another, a large bone called the os calcis, forms the heel posteriorly.

The metatarsal bones are five in number, and are longer and more slender than the metacarpal bones. They articulate with certain of the tarsal bones behind, and with the first phalanges in front.

The phalanges, as in the hand, are three in number to each toe, except the great toe, which has only two.

The bones of the foot form a very beautiful arch from before backwards. The posterior pillar of this arch is short, and consists of the os calcis and part of the astragalus; while the anterior is longer, and is made up of some of the other tarsal bones with the rounded heads of the metatarsal bones, upon which, and the heel, we walk. This arch contributes largely to the elasticity of the foot. (See Fig. 10.)

### The Humanising of the Poor Law.

The circular which the Local Government Board has this week issued to Boards of Guardians throughout the country draws attention to the desirability of certain points of workhouse administration being duly considered. Many of the recently-elected Guardians having had no previous experience in the administration of the Poor Law, it is deemed fitting that they should be asked to realise "that arrangements originally adequate, and in accordance with the spirit of the times, have ceased to be so," owing to the character of those for whom accommodation is provided having materially changed. A general order last year gave authority to every member of a Board of Guardians to visit the workhouse at any reasonable time that he might think proper; and the Local Government Board considers surprise visits of great value in ascertaining the real character of the administration of a workhouse.

The Guardians are also expressly empowered to appoint committees of ladies "whose duty it should be to visit and examine those parts of the workhouse in which the female inmates and the children are maintained, and to report to the Guardians any matters which may appear to the committee to need attention."

The circular also deals with the question of the nursing of the sick, with the adequate remuneration of nurses, and with the obvious disadvantages attaching to the employment of pauper assistants in infirmaries. "The Guardians should be satisfied that the nursing staff by day and by night is in numbers fully equal to the proper nursing of the sick, and they should give their most careful consideration to any representations which may be made to them on the subject by the medical officer. . . . They should also be careful when they make appointments of nurses that the persons appointed are, by training and experience, fully equal to the responsible duties which they have to discharge."

As long as the present constitution of the workhouses remains unchanged the nurses are responsible to the medical officer for the treatment of the patients, and in other matters they are under the authority of the master and matron. It is evident that the Local Government Board duly realises the importance of all matters relating to the moral and physical well-being of the children for whom the Poor Law is responsible, as the present circular especially commends these matters to the notice of the Guardians.

It should be pointed out that though the circular is just issued it really contains no new regulations. All these things have been within the guardians' power for a considerable time, and we are glad to say that in many unions all of these suggestions have long been acted on. But in many they have not, and it is to be hoped that this official reminder of the gentler side of poor-law guardianship will tend greatly to the humanising of the workhouses in many dark places.

### Minor Appointment.

Cottage Hospital, Warminster.—Miss E. Garnett Clarke has been made Nurse-Matron at this hospital. She was trained at the Children's Hospital, Pendlebury, and Royal Southern Hospital, Liverpool, and was afterwards a sister at the latter hospital; also one of Her Majesty's Nursing Sisters; and district nurse at Nailsea, Somerset.

Miss A. Walliscraft has been appointed Sister of the male wards in the Oldham Infirmary, in which hospital she trained for three years, and at the completion of her training, two years ago, she left to take the post of Sister of female wards in the Chichester Infirmary. In being appointed Sister in the Oldham Infirmary she has the good wishes of, and a hearty welcome from, all her old friends.

### Notes from Germany.

Homes for Consumption.

Professor Leyden's lecture, delivered at the International Congress held this year at Buda-Pesth, on "The Accommodation and Nursing Provided for Tuberculous Patients in large Towns," appears in full in the last number of the *Zeitschrift für Krankenpflege.* Speaking of the spread of a disease which carries off a greater number of victims than cholera or diphtheria, the Professor estimated the death-rate from it in Prussia in the past year to be 88,000. In Berlin, in the same year, 3,800 persons died of consumption, the number of persons suffering from the disease in that city being 25,000, belonging chiefly to the lower classes and in the prime of life. Professor Leyden describes the different institutions erected for the benefit of such patients. Dr. Brehmer, in Görbersdorf, is the physician to whom Germany is indebted for the erection of the first. According to the last report issued by the heads of the Falkenstein Institution, restorations to health average 24 per cent. Herren Drs. Dettweiler and Finkleberg were instrumental in its erection. It was opened on August 13th, 1892, by the Empress Frederick, and contains 28 beds. During the first year 133 in-patients were treated, 102 being discharged in improved health. The treatment generally extended over 71 days, the increase of weight per patient amounting to something like 7 lb. The inclusive cost for each patient was M2·50. Dr. Dettweiler, the medical superintendent, is assisted by two other doctors.

Germany is indebted to the Frankfort *Verein für Reconvalescenten-Anstalten* for the erection of a larger Home, which is in course of construction, and will afford accommodation to 70 or 80 patients of either sex at Rupertshain, near Königstein. It will be opened in the early spring of next year.

The city of Worms has also a similar project in hand at Felsberg in Odenwald, which will provide accommodation for thirty-five patients. On July 1st, 1893, a Home, erected by the Bremer Verein, was opened at Rehburg in the Harz Mountains. Under the patronage of His Majesty the King of Saxony, Dr. Driewer has organised a committee to arrange for the erection of a sanatorium spacious enough to receive from 100 to 120 patients at Rehboldsgrün. The estimated cost of the building is M.250,000 (£12,500), which is to be defrayed by voluntary donations. Dr. Ladendorf, of Andreasberg, is also at the head of a Home supported by charity.

Professor Leyden described briefly similar institutions in England and other countries. Vienna is to be congratulated upon the splendid gift of Baron V. Rothschild of a castle, surrounded by a luxuriant park, which is now being converted into a Home for tuberculous patients.

An institution was also opened in 1892 at Malchow, near Berlin, which affords accommodation for 96 patients, but neither its situation nor construction are favourable for its present use.

Professor Leyden concluded by advocating the employment of the patients, both for their healths' sake and also as a matter of economy; he likewise emphasizes the importance of not admitting those who are in such an advanced stage as to render treatment unavailing.

The "Nerven Klinic."

Professor Flechsig, the director of the "Nerven Klinic" at Leipzig, may well feel proud of his institution, which is a large handsome building situated on the outskirts of the town and surrounded by spacious grounds and gardens, and overlooking fields. It is approached by a well kept drive, and the buildings are raised from the ground by a flight of stone steps. On the left is the porter's room, the windows of which overlook the entrance, enabling him to keep count of all who enter or leave the institute. The entrance hall is spacious aud lofty, the white stone pillars having an imposing appearance. The right wing is apportioned to the accommodation of the male patients, male attendants, and house surgeon, Dr. Teuscher. The latter merits the esteem in which he is held for the kindly interest he takes in his patients, rich and poor alike. The female inmates, their attendants, and special surgeon are accommodated in the left wing. The windows of all the patients' rooms overlook the beautiful grounds, and a terrace at the back of the building is reserved for invalids who lie there on comfortable sofas or reclining chairs every sunny day. A prettily fitted up chapel forms part of the buildings, where all who are willing and able attend service on Sundays. There is a large billiard-room, and other amusements are provided for the patients. The diet is good and abundant, and the patients are visited by their friends on Wednesday and Sunday afternoons from two to four, and on any other day by permission of the house surgeons. Professor Flechsig resides in a detached house within the grounds, and he supervises the treatment of the patients. He has made mental diseases his especial study, and his large experience and keen observation fit him to fill the high post which he has so long occupied.

### The Book World for Women and Nurses.

[We invite Correspondence, Criticism, Enquiries, and Notes on Books likely to interest Women and Nurses. Address, Editor, The Hospital (Nurses' Book World), 428, Strand, W.C.]

### MAGAZINES OF THE MONTH.

The Pall Mall Magazine keeps up its character for useful and entertaining reading, the February number being no exception to the rule. The editor contributes a paper on spiritualism; he is evidently no believer in table-turning and spirit-rapping, and while he grapples with the subject from a very clear common-sense point of view, it will probably not find much favour from the numerous lovers of the supernatural. Mr. Besant winds up his interesting chapters on Westminster and its environs, and we sigh for the Sunny South, with its beauties of nature and art, as we read "Florentine Pictures," by Charles Godfrey Leland. Anything about China is acceptable now, so that "Looting the Summer Palace," by China Jim, will no doubt be read with interest. Instead of prairies and orange groves we are transported to Tasmania and get an account of "Apple Land" from Mr. Macnaghten's able pen, the illustrations of which are excellent. The account is quite sufficiently interesting to perhaps induce some enterprising spirits to go out and see if anything can be made out of the production of apples as well as oranges. The magazine also contains a number of poems and stories by well-known authors, Arthur Symons, Rider Haggard, Phil Robinson, and others. Not the least among the tales is one entitled "Colombe," by Minnie Buchanan Goodman; it is charmingly told and illustrated fisher-life in Normandy.

We are not disappointed in the English Illustrated for February, it is a capital number, with a variety of matter which will doubtless suit all readers. Stanley J. Weyman still continues his entertaining "Memoirs of a Minister of France." It is pleasant to hear anything of Henri of Navarre, and the memoirs throw a great light on the manners and customs of the times. Those who care for antiquarian research will appreciate the paper entitled "Two Dozen Greek Coins," by Edward L. Cutts. "The Man and the Town," by Frederick Dolman, takes us to Yarrow-on-Tyne. In the old days Yarrow and Bede were invariably associated, now, at any rate in the commercial mind, it is Yarrow and Palmer. Another "Moorland Idyll" by Grant Allen! How charming these idylls are; how he idealises everything he touches—only "a flight of quails," yet full of poetic beauty and imaginative thought! The papers, "How the Other Half Lives," are still continued, "The Policeman" is the subject this time, by Mr. Wilfred Wemley. There are also a variety of stories in the magazine which are wisely adapted for the taste of a variety of readers.

### Notes from St. Helena.

By Rose A. Blennerhassett, Lady Superintendent of the Civil Hospital.

### IV.—MISTAKEN ECONOMY.

It appears that the Governor, strongly objecting to the existing organisation, had for a long time urged the necessity of having a certificated nurse to take charge of the hospital, but had been unable to carry his point until the autumn of 1893. The plan of leaving the hospital practically without an acknowledged head was an exceedingly "penny wise and pound foolish" one. The amount spent on stimulants alone has decreased from £39 to £9 16s. 4d. for an equal number of patients in a given time, by which some idea may be formed of the waste which prevailed, without taking into consideration the ruin of instruments and destruction of bedding, through the want of the simplest precautions.

At first it might be believed that the "cook nurse" and orderly system was a cheap one, at least as regarded salary, but on finding that they divided between them a sum, which with allowance for night work, amounted to about £114 a year, no time was lost in begging the Governor to make a new arrangement and have a trained nurse. His Excellency consented, and Sister Lucy Sleeman undertook to fill the post until a nurse could be obtained from Europe or from Kimberley.

### Pleasant Nursing Posts.

So we are the first nurses who have been in St. Helena, and we like to think that we shall leave behind us a fairly creditable hospital, as well as two nursing posts, which are by no means to be despised. They are well paid, the life is pleasant, the work light—too light as a rule—but heavy now and then, at more or less long intervals. Rushes of work depend chiefly on the health of whaling and coolie ships which touch here, any passing ship with a sick man on board generally stopping to land him.

The hospital is managed on the paying system, the Government making up any deficit. Islanders pay 1s. a day; sailors, 3s. a day. The parish pays 9d. a day for each pauper patient. There is also a charitable fund, which pays for 600 diets, at the rate of 9d. a day.

This charity had a curious origin. A certain lady had a lover, who "loved and rode away." Her father caused an action for breach of promise to be brought against him, won his suit, and devoted the damages to the Civil Hospital.

The islanders generally belong to different associations and clubs, to which they regularly subscribe, the association paying hospital fees, and giving outdoor relief to men unable to work. Sometimes we wonder whether a similar system of small payments would not be an advantage at home. Might it not do something towards lessening that abuse of hospital charities of which one is beginning to hear so much?

### Napoleon's Tomb and Favourite Well.

Of course, we visited Napoleon's Tomb, and equally, of course, were disappointed! Where was the willow beneath which he used to rest when alive, and which overshadowed his grave? Long since dead, we were told. A circle of tall dark trees, mostly firs, fringe a grassy hollow, in whose depths lies a great grey slab, fenced in with iron railings. Close by, a spring of clear, cold water bubbles up. This was the only water which the exile would drink, and visitors are expected to taste it. An atmosphere of tranquil melancholy surrounds this cool, green spot, which contrasts sharply with barren, desolate Longwood, a place hateful to Napoleon.

### Longwood.

And yet Longwood has a beauty of its own—with its wide-reaching view over seemingly limitless ocean, and the wonderful lights and shadows that play over its rocky heights. The air, too, is fresh and bracing on the hottest day.

Napoleon much preferred a charming châlet near Jamestown, called the Pavilion of the Briars, which is surrounded by the most luxuriant vegetation. He spent six weeks in it, but it was considered to be too near the port for safety, and he was removed to Longwood. Before coming to St. Helena our impression was of an arid desolate rock, and from the sea it has this appearance. But no place in the world, of equal size, can boast of such lovely and varied scenery. The transitions from stony wilderness to the most lavish wealth of greenery are abrupt and unexpected.

### Everybody's Opinion.

[Correspondence on all subjects is invited, but we cannot in any way be responsible for the opinions expressed by our correspondents. No communications can be entertained if the name and address of the correspondent is not given, or unless one side of the paper only be written on.]

### SPURIOUS CO-OPERATIONS AND HOMES.

"A Private Nurse" writes: The letter in a recent issue from "A Well Wisher of The Hospital" described accurately a so-called "co-operation and home," to which I once had the misfortune to belong for some time, and were it not for a few nurses who remain at the place—for reasons best known to themselves—I would gladly furnish you with the address for publication in your valuable and widely read paper, and so close this "snare and delusion." In no sense is it either "co-operation" or "home."

### The Hostel of God.

The establishment founded under this title by S. James' Servants of the Poor has published its report, which acknowledges the assistance of the hon. secretary, Captain Portlock Dadson, by whose intervention an office has been secured without rent at 281, Strand. A naval, an army, and a Masonic bed have been instituted, and the Sisters are hopeful of adding others to their number. Special mention is made of the liberality with which certain nurses have freely given their services to the incurable cases which the hostel expressly treats in the ten beds to which its present accommodation is limited at 82, The Chase, Clapham. An appeal for increased subscriptions to enable the Sisters to double the number of beds is now made, and to this should be added a recommendation that a general and an executive committee be also appointed.

### Notes and Queries.

The contents of the Editor's Letter-box have now reached such unwieldy proportions that it has become necessary to establish a hard and fast rule regarding Answers to Correspondents. In future, all questions requiring replies will continue to be answered in this column without any fee. If an answer is required by letter, a fee of half-a-crown must be enclosed with the note containing the enquiry. We are always pleased to help our numerous correspondents to the fullest extent, and we can trust them to sympathise in the overwhelming amount of writing which makes the new rules a necessity. Every communication must be accompanied by the writer's name and address, otherwise it will receive no attention.

### Queries.

(70) *Cripple.*—Where can I find particulars of the treatment suggested by Lorenz for dislocation of hip?—*Auntie.*

(71) *Nurses' Library.*—Where can I get a list of technical books suitable for a nurses' library?—*Subscriber.*

(72) *Abbotsford Tweed.*—Where should I write for this tweed, noticed in The Hospital.—*Enquirer.*

(73) *The Kimberley Hospital.*—The Matron asks if there is any agent or person in England of whom she can make inquiries about the conditions of appointment of matron to the Kimberley (South Africa) Hospital. She fears there is not time to write by mail and receive an answer.

### Answers.

(70) *Cripple (Auntie).*—In the *Wiener Medical Presse*, No. 11, 1892, Lorenz describes his new procedure for cases of congenital dislocation of the hip. There is a short abstract in the Epitome of the *British Medical Journal*, May 6th, 1893.

(71) *Nurses' Library (Subscriber).*—Write to the Scientific Press, 428, Strand, for catalogue.

(72) *Abbotsford Tweed (Enquirer).*—Write to Abbotsford Tweed Company, Galashiels, N.B.

(73) *The Kimberley Hospital (Matron).*—Matters are most unsatisfactory, and we hope to see the early appointment of a Government Commission to inquire into the management and suggest reforms. Till this is done English gentlewomen had better not apply for the vacant post.

### Two Hours Off Duty.

### ROUND ST. BARTHOLOMEW'S HOSPITAL.

St. Bartholomew's is within three minutes' walk of many interesting remains of mediæval London. The only difficulty is to decide to which historic spot we will direct our steps during the limited space of time at our disposal. Smithfield has an ugly sound in the ears of all Englishmen; it recalls, however, one of the best *bona fide* examination stories we have heard for some time, and which is at least more amusing than stories of burning humanity. A promising student of history stated that "After the death of Edward VI. the unfortunate Mary Queen of Scots ascended the throne, assuming the title of 'Bloody'!"

The noble church founded by Rahere is too well known to detain us long; it is one of the earliest examples of church architecture in England after the introduction of the chisel among the Norman builders, and accordingly we find there some of the typical ornaments which, later in the style, were developed with such effect by architects who had been to the Crusades. In the East men saw the Byzantine method of church decoration, and upon their return to Western Europe executed the rich surface sculpture in imitation of it, which is the grand feature of 12th century architecture.

In the ambulatory of St. Bartholomew the Great will be found what is called a barrel vault; it has no claim to beauty certainly, being clumsy and heavy looking, yet some archæologists would rather watch the destruction of the pendants in Henry VII.'s Chapel than lose this rude early effort of the builder to span an aisle with a vaulted roof.

The Smithfield markets are not savoury to-day, a far pleasanter place for a walk is the quiet, old-world seclusion of Charterhouse Square. The Barbican close by enables us to realise that this region once lay outside the City walls, and it may have a professional interest for nurses to know that the neighbourhood is associated with a great epidemic.

About twenty-three years before the Charterhouse was built the Black Death was ravaging England. This pestilence, which has been the subject of much scientific and historical research lately, was a potent factor in social evolution, breaking through the fetters of feudalism and strict ecclesiastical discipline with momentous results, good and bad, for succeeding generations, but requiring very extensive graveyards for the reception of its victims while it lasted. Ralf Stratford, Bishop of London, bought and consecrated a piece of ground just outside the City wall, about West Smithfield. Here he built a mortuary chapel, calling the place the Pardon Churchyard and Chapel, but even this space was insufficient to meet the demands for burials, and Sir Walter de Mauny added the adjoining St. Bartholomew's Spital, which, as a cemetery, became known as New Church Hawe. These acres, with a further purchase of land from the adjacent Hospital of St. John of Jerusalem, comprised the domain of the "Prior and Convent of the House of the Salutation of the Mother of God," commonly called the London Charterhouse, which was founded by Sir Walter de Mauny and Bishop Michael de Northburgh.

The halls of most of the City companies are worth a visit, and none of them are far from St. Bartholomew's Hospital. The Great Fire, indeed, destroyed nearly all the original buildings, but the records, deeds, and old plate of these guilds repay one for very considerable study. The various corporations in the middle ages held commercial integrity, charitable deeds, and religious observances in veneration besides recognising the duties of hospitality, as the following examples will show: In 1456 the Grocers' Company fined one, John Aysfield "vi s. viij d." for "offens don in makyng of untrewe powder, gynger, cynemon, and sanaders"; while it is recorded of the Goldsmith's in Richard I.'s reign, finding that many persons of that trade "lost their sight, became crazed and infirm" from the ill effects of the fire and smoke of quicksilver necessary in their trade, they "divers of the said company compassionating the condition of such" gave tenement rents to the "value of twenty pounds per annum" for their maintenance, and "of a chaplain to celebrate mass amongst them every day for the souls of the faithful departed."

Some of the City companies had "sisters" attached to them as well as brothers or masters, and wives and daughters were not only admitted to the feasts in some corporations, but the brethren were even fined for not bringing their women folk to partake of the good cheer. The list of boiled capons, roasted swans, salmon, wafes, and other good things provided for the ladies, in proportion to their rank and importance, at an election feast of the Drapers' Company in 1515 has survived; the higher the social status the larger the appetite, seemed to be this company's experience with regard to its feminine guests, and "maydens" were not expected to eat as much as great ladies.

Two valuable relics of Old London are preserved in the very heart of the new, so we will pass the row of houses, behind which the Merchant Tailors' Company still has its home, and make for Bishopsgate Street. In the Merchant Tailors' kitchen eighteen haunches of venison can be cooked at once, and the Tailors' have, moreover, a small crypt left of their old building, destroyed in the Great Fire; but a far more interesting building is Crosby Hall, in Bishopsgate Street, one of the most beautiful mediæval dwellings remaining in London. Sir John Crosby was a "grocer and woolman"; he was a City magnate in the fifteenth century, and was knighted by the merchant king, Edward IV. He obtained a lease of this property from the Prioress of St. Helen's, the beautiful church of which conventional establishment still stands just behind Crosby Hall, and from the interesting tombs it contains has been called the "Westminster Abbey of the City."

We have not space to tell the story of the vicissitudes of Crosby Hall, but one owner, Sir John Spencer, in Tudor times, had a daughter and heiress who was courted by the Earl of Northampton contrary to the desire of her father. It is related that one day Sir John met a baker's boy with a covered barrow at his door, and that the knight even tipped him with sixpence, but when he discovered that the baker's boy was the disguised Earl of Northampton, and that the barrow contained Mistress Elizabeth Spencer, he vowed that sixpence should be the only money of his his son-in law ever handled. However, a year afterwards Sir John Spencer was invited by Queen Bess to be fellow gossip with her to a new-born baby friend of hers. Naturally, the invitation was a command, and the baby settled the quarrel between Sir John and its father and mother, so that the property came to the Comptons after all, and an amusing memorial of the story remains in the Spencer monument in St. Helen's Church to this day.

Concerning this church of Great St. Helen and the Holy Rood, a book might be, and indeed has been, written. Here are survivals of the Romans and of the nuns, a "squint" which we believe to be unique in character, many very fine pre-reformation tombs, and an altar stone with its five crosses, some good window tracery and execrable glass, brasses worth half an hour's work with some heelball, and a queer little figure of a young lady reading, which some unaccountable tradition in the face of common sense asserts to be intended for St. Helena.

Our two hours would seem like two minutes, however, if we once get within the precincts of this church, which the incumbent clergyman will be happy to show us himself when our "long day" happens to fall on a Wednesday, if we can attend the Litany in the morning, and remain for an hour after it is over. And surely this trouble is not too much to take in order to see a church which, were it only in France instead of at our doors at home, many English people would be anxious enough to visit.

## Figures and Tables

**Fig. 8. f1:**
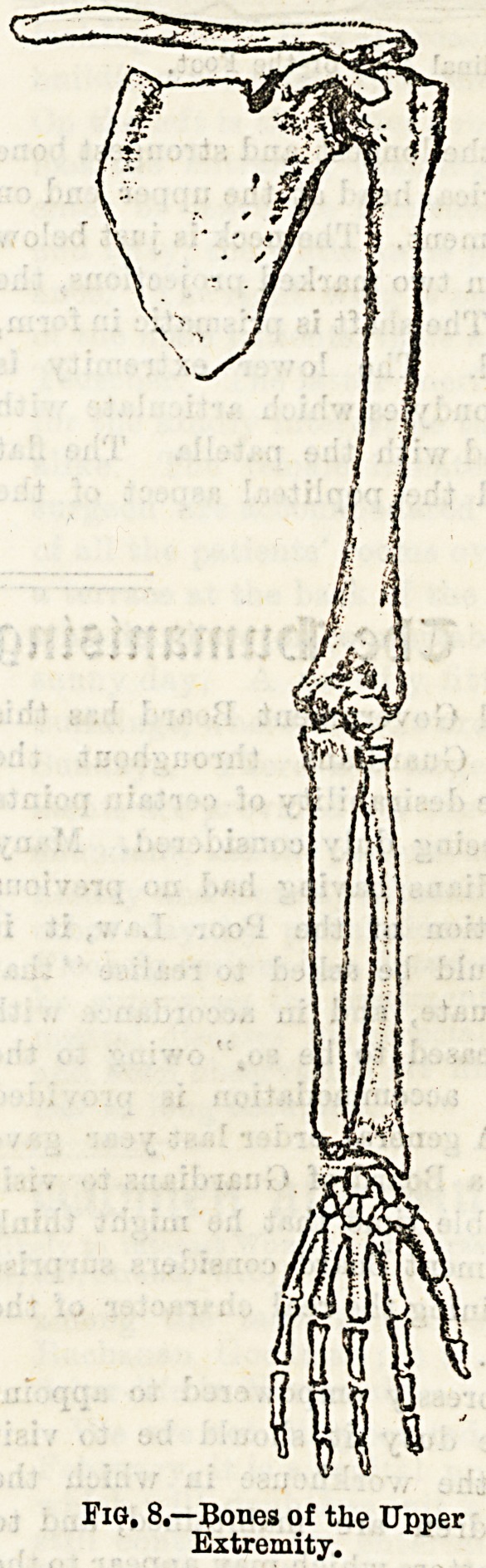


**Fig. 9. f2:**
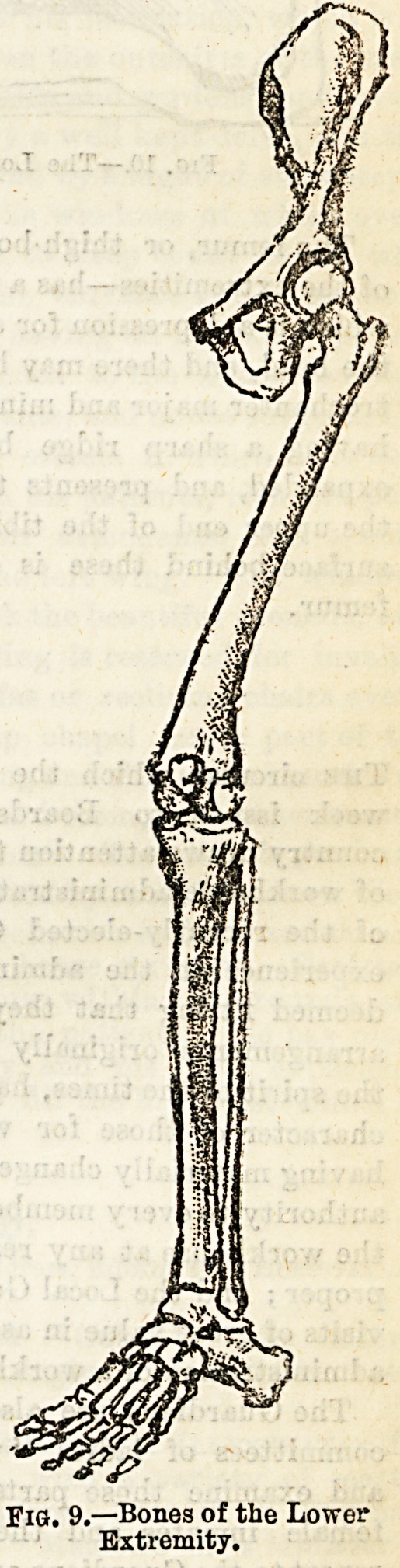


**Fig. 10. f3:**